# The evaluation of playing styles integrating with contextual variables in professional soccer

**DOI:** 10.3389/fpsyg.2022.1002566

**Published:** 2022-09-23

**Authors:** Lingfeng Kong, Tianbo Zhang, Changjing Zhou, Miguel-Angel Gomez, Yue Hu, Shaoliang Zhang

**Affiliations:** ^1^Department of Physical Education, Hohai University, Nanjing, China; ^2^Department of Automation, Tsinghua University, Beijing, China; ^3^School of Physical Education and Sports Training, Shanghai University of Sport, Shanghai, China; ^4^Faculty of Physical Activity and Sport Sciences (INEF), Universidad Politécnica de Madrid, Madrid, Spain; ^5^Department of Political Science, Tsinghua University, Beijing, China; ^6^Division of Sports Science and Physical Education, Research Centre for Athletic Performance and Data Science, Tsinghua University, Beijing, China

**Keywords:** styles of play, principal component analysis, soccer, match performance, team sports

## Abstract

**Purpose:**

Playing styles play a key role in winning soccer matches, but the technical and physical styles of play between home and away match considering team quality in the Chinese Soccer Super League (CSL) remain unclear. The aim of this study was to explore the technical and physical styles of play between home and away matches integrating with team quality in the CSL.

**Materials and methods:**

The study sample consists of 480 performance records from 240 matches during the 2019 competitive season in the CSL. These match events were collected using a semi-automatic computerized video tracking system, Amisco Pro^®^. A k-means cluster analysis was used to evaluate team quality and then using principal component analysis (PCA) to identify the playing styles between home and away matches according to team quality. Differences between home and away matches in terms of playing styles were analyzed using a linear mixed model.

**Results:**

Our study found that PC1 presented a positive correlation with physical-related variables such as HIRD, HIRE, HSRD, and HSRE while PC2 was positively associated with the passing-related variables such as Pass, FPass, PassAcc, and FPAcc. Therefore, PC1 typically represents intense-play styles while PC2 represents possession-play styles at home and away matches, respectively. In addition, strong teams preferred to utilize intensity play whereas medium and weak teams utilized possession play whenever playing at home or away matches. Furthermore, the first five teams in the final overall ranking in the CSL presented a compensated technical-physical playing style whereas the last five teams showed inferior performance in terms of intensity and possession play.

**Conclusion:**

Intensity or possession play was associated with the final overall ranking in the CSL, and playing styles that combine these two factors could be more liable to win the competition. Our study provides a detailed explanation for the impact of playing styles on match performances whereby coaches can adjust and combine different playing styles for ultimate success.

## Introduction

There is a universal phenomenon in the world of football commentary and coaching that refers to how a game “unfolds” or what playing styles are typically executed in the competition (Hewitt et al., 2016). The culture of soccer clubs may refer to styles of play with colloquiums such as “Total Football” or “Tika-taka.” It is thus clear that a style of play could be considered as the general behavior of the whole team to achieve the attacking and defensive objectives during the match (Zhang et al., 2019). Therefore, coaches and coaching staff should be aware of the different contexts where various playing styles occur based on each phase of the game to better adjust strategies and tactics and improve match performance.

Playing styles have been identified in different national soccer leagues using principal component analysis (PCA) as a common and robust method to extract the main components of the team’s performance, and then verify the playing styles of the teams in the respective soccer league (Pino-Ortega et al., 2021). Specifically, Castellano and Pic (2019) investigated the twenty teams from the Spanish first division in the 2016–2017 season using nine interaction performance indicators. This study found that deep or high-pressure defending, and elaborate or direct attack were the key winning factor based on the first two PCs. Furthermore, Gómez et al. (2018) reported that extracting eight factors allowed to identify playing styles according to team quality and match location. This study found that ball possession and shot-related variables, defined by PC 1 and PC 2, were the most apparent styles in Greek professional soccer. Likewise, Gollan et al. (2018) identified that match success for the top-ranked team was associated with dominance in transition moments, and playing styles vary across teams but are associated with the final ranking position in the English Premier League. In addition, the previous studies investigated the 380 matches of the 2015-2016 English Premier League season and pointed out that contextual variables must be considered in future studies when quantifying the styles of play in elite soccer because match status, match location, and quality of opposition influence playing styles (Fernandez-Navarro et al., 2018, 2019).

Optimizing match performance in the Chinese Super League (CSL) has received widespread attention in recent years. The related studies have largely focused on the strategy of ball possession (Liu et al., 2021), the evolution of performance indicators (Zhou et al., 2020), and the influence of contextual variables on match performance (Gai et al., 2019; Zhou et al., 2021a). Particularly, several technical variables such as foul, pass, air duel, tackle, shot, and corner kick and physical variables like sprint and high-speed running distance were associated with ball possession in the CSL (Gong et al., 2021). In addition, performance variables generally showed significant upward trends from the 2012 to 2017 seasons in the CSL, specifically, there were ∼23% more crosses, ∼12% more shots on target, and ∼11% more opponent penalty area entries (Zhou et al., 2020). Furthermore, superior teams at home have better performance in terms of shot, shot on target, shot off target, penalty, and shot from the outside box, while visiting teams tend to take a more stable strategy (Liu et al., 2019). Likewise, shot on target, shot accuracy, cross accuracy, tackle, and yellow card were the five key performance indicators that showed consistent effects on winning matches; other effects varied depending on the strength of the team and opposition (Zhou et al., 2021a).

The results of the aforementioned investigations are difficult to be applied to football teams as it is hard and non-contextualized to train all the factors associated with performance. From a practical perspective, the application of a specific playing style is a simpler way to increase the rating of performance indicators within a team (Lopez-Valenciano et al., 2021). To the best of our knowledge, there are only two studies (Lago-Peñas et al., 2017; Zhou et al., 2021b) exploring the playing styles of teams, but one study only selected technical variables to evaluate playing styles and another one added physical variables to explore the evolution of styles of play without controlling for situational variables in the CSL. Therefore, this study aims to explore the styles of play between home and away matches integrating with team quality in the CSL. We hypothesized that there could be a considerable difference in terms of playing styles for each team between home and away matches in the CSL.

## Materials and methods

### Subjects

The study sample consisted of 480 performance records from 240 matches during the 2019 competitive season in the CSL. These match events were collected using a semi-automatic computerized video tracking system, Amisco Pro^®^. The validity and reliability of this system have been verified in previous studies (Carling et al., 2008; Castellano et al., 2014).

### Procedures

Based on previous literature (Lago-Peñas et al., 2017; Gong et al., 2021; Zhou et al., 2021b), nine physical performance-related parameters, 12 technical performance-related parameters, and two situational variables were chosen for the analysis. The categories and definitions of these variables are presented in Table 1. The speed thresholds of the physical performance parameters were similar to that of the previous report (Bradley et al., 2009, 2016). The ethics committee approval for this study was obtained from the Shanghai University of Sport.

**TABLE 1:** Category and definition of the technical and physical variables.

**Abbreviation – Physical performance-related parameters (unit): operational definition**

**TD** – **Total Distance (m):** distance covered in a match by all the players of a team. **SprintD** – **Sprint Distance (m):** distance covered at a speed of over 25.1 km/h in a match by all the players of a team. **SprintE** – **Sprint Efforts:** number of sprints (speed > 25.1 km/h) in a match by all the players of a team. **HSRD** – **High-speed running distance (m):** distance covered of high-speed (19.7–25.1 km/h) running in a match by all the players of a team. **HSRE** – **High-speed running efforts:** number of high-speed (19.7–25.1 km/h) running in a match by all the players of a team. **HIRD** – **High-intensity running distance (m):** High-intensity running consisted of running, high-speed-running, and sprinting (running speed > 14.4 km/h). **HIRE** – **High-intensity running efforts:** number of high-intensity running in a match by all the players of a team. **MSRD** – **Moderate-speed running distance (m):** distance covered at moderate-speed running (14.3–19.7 km/h) in a match by all the players of a team. **LSRD – Low-speed running distance (m):** distance covered at low-speed running (7.1–14.3 km/h) in a match by all the players of a team.

**Abbreviation** – **Technical performance-related parameters (unit): operational definition**

**Shots:** attempts to score a goal made with any (legal) part of the body, either on or off target. **ShotAcc** – **Shot Accuracy (%):** shots on the target as a proportion of the total shots. **Passes:** intentional played balls from one player to another. **BP** – **ball possession (%):** The duration when a team takes over the ball from the opposing team without any clear interruption as a proportion of total duration when the ball was in play. **PassAcc - Pass Accuracy (%):** successful passes as a proportion of the total passes. **FPass** – **Forward Passes:** intentional played balls from one player to another who is located in the opponent’s half of the pitch. **FPAcc** – **Forward Pass Accuracy (%):** successful forward passes as a proportion of the total forward passes. **Challenges:** actions when two players are competing for ball possession, which is not in control of any player, i.e., both players have approximately 50% chance of gaining control of the ball; includes ground and air challenges. **ChallengeW** – **Challenge Won (%):** successful challenges as a proportion of the total challenges. **Fouls:** any infringement penalized as foul play by a referee. **Corner:** ball goes out of the play for a corner kick. **Offside:** being caught in an offside position resulting in a free kick to the opposing team.

**Situational variables: operational definition**

**Match location:** venue of the match—playing at home or away. **Team quality:** competitive level of a team was evaluated by cluster analysis. A team was classified as “strong” (ranking from the 1st to 5th place), “medium” (ranking from the 6th to 11th place), and “weak” (ranking from the 12 to 16th place).

The unit of the physical and technical performance-related parameters without units are in counts.

### Statistical analysis

First, the k-means clustering algorithm was used to separate teams into different clusters based on the final ranking positions in the CSL. The optimal number of clusters was determined upon visual inspection of a scree plot (i.e., the elbow method) whereby the highest number of clusters that reduced the within-cluster variation substantially was identified (Zhang et al., 2017, 2018; Shelly et al., 2020). The results identified three clusters as follows: cluster 1 (Strong, final ranking in the league from 1st to 5th), cluster 2 (Medium, final ranking in the league from 6th to 11th), and cluster 3 (Weak, final ranking in the league from 12th to 16th).

Second, the central idea of the principal components analysis method (PCA) is to reduce the dimensions of data that have a large number of interrelated variables while preserving the maximal variance (Abdi and Williams, 2010). Specifically, the principal component analysis consisted of the calculation of eigenvectors and eigenvalues from the covariance matrix of *M* (O’Donoghue, 2008). Eigenvectors are the vectors of coefficients corresponding to eigenvalues and were used to calculate the results (Nguyen and Holmes, 2019). Thus, the coefficients represent the loading factors of each original variable to obtain the newly transformed data, and the positive or negative value represents a direct or inverse proportionality, respectively (Weaving et al., 2019). Finally, the original data were subsequently projected onto the eigenspace of the covariance matrix which provided the PC scores (Abdi and Williams, 2010). Furthermore, in order to perform principal component analysis (PCA), our study first examined the data for suitability by Bartlett’s test of sphericity and the KMO measure of sampling adequacy. Specifically, Bartlett’s test of sphericity was computed to provide the statistical significance that the correlation matrix has significant correlations among at least some of the variables. The measure of sampling adequacy was also developed with Kaiser-Meyer-Olkin (KMO) and computed to evaluate the appropriateness of applying factor analysis, considering that values above 0.50 for the entire matrix or an individual variable indicate appropriateness. In addition, the number of PCs to be retained was based on eigenvalues (greater than 1.0) and that explained higher than 60% of the percentage of variance. Furthermore, although factor loadings of ±0.30 to ±0.40 are minimally acceptable, values greater than ±0.60 were considered for practical significance (Gonçalves et al., 2019).Notably, the first and second PCs were extracted according to the previous studies since they explain the most amount of variance in the dataset (McCormack et al., 2021; Racinais et al., 2021) and 2 PCs were required to identify the individual and variable responses in a two-dimensional space as well as visualize the playing styles of each team between home and away matches in the CSL (Weaving et al., 2019; Lopez-Valenciano et al., 2021).

Third, linear mixed models were used to assess differences in terms of playing styles between home and away matches considering team quality. The variables of match and team were regarded as random effects while contextual variables (i.e., match venue and quality of opposition) were the fixed effects in the models. The assumptions of homogeneity and normal distribution of the residuals were also verified for each model. Pairwise comparisons between different levels of teams were conducted *via* Bonferroni adjusted *post hoc* test (Lago-Peñas et al., 2022). Effects sizes (ES) were calculated using Cohens’ d according to the formula d = (M2 – M1/SDpooled), where M1 and M2 are the means of the two groups and SDpooled is the square root of the weighted average SD of each group. Values greater than or equal to 0.2, 0.5, and 0.8 were considered to represent small, medium, and large differences, respectively (Fritz et al., 2012).

All analyses were conducted using the statistical programming environment R (version 4.1.2). Specifically, the k-means clustering algorithm was performed using the “*kmeans*” function from the “*stats*” R package; principal component analysis (PCA) was conducted by the “*factoextra*” and “*FactoMineR*” package; linear mixed model and *post hoc* tests were performed using “*lme4*” and “*emmeans*” package. Visualization of difference was conducted according to the “*ggstatsplot*” package. *P* < *0.05* was considered statistically significant.

## Results

The principal components model accounted for 55% of the total variance for home matches and 54.2% of the total variance for away matches in Table 2, with the first and second component factors extracted for home and away matches. It is worth noting that the first components mainly consisted of HIRD, HIRE, HSRE, HSRD, SprintE, SprintD, and TD while the second highlighted FPAcc, PassAcc, FPass, Pass, and BP at home and away matches in Table 2.

**TABLE 2:** Component factor loadings, component statistics, Bartlett’s test of sphericity and Kaiser-Meyer-Olkin measure of sampling adequacy of the factor analysis (principal component methods) between home and away matches.

Variables	Component factors (Home match = 240)					Component factors (Away match = 240)				
	1	2	3	4	5	1	2	3	4	5
BP	0.223	**0.776**	–0.271	–0.110	–0.250	0.325	**0.782**	0.207	0.131	0.264
Foul	–0.007	–0.351	–0.085	0.329	–0.461	–0.075	–0.353	0.139	0.193	0.502
Corner	0.354	0.145	–0.336	–0.515	0.158	0.137	0.259	0.445	0.531	–0.342
Offside	0.055	–0.081	–0.269	0.425	0.031	0.181	0.121	0.182	–0.124	0.246
Shot	0.441	0.363	–0.300	–0.365	0.201	0.212	0.396	0.438	0.300	–0.336
ShotAcc	0.118	–0.153	0.002	**0.612**	0.383	–0.091	–0.014	–0.104	–**0.671**	0.444
Pass	0.367	**0.869**	0.147	0.035	–0.159	0.424	**0.856**	–0.116	0.059	0.155
PassAcc	0.176	**0.909**	0.094	0.183	0.088	0.197	**0.895**	–0.116	–0.231	–0.009
FPass	0.401	**0.832**	0.121	0.015	–0.194	0.447	**0.823**	–0.074	0.107	0.182
FPAcc	0.217	**0.903**	0.042	0.159	0.118	0.234	**0.902**	–0.042	–0.206	–0.015
Challenge	0.121	–0.371	–0.068	–0.574	–0.345	0.134	–0.276	0.142	0.546	0.499
ChallengeW	0.076	–0.035	0.116	–0.234	**0.607**	–0.085	–0.024	0.254	–0.356	–**0.672**
TD	**0.742**	–0.166	**0.619**	–0.052	–0.012	**0.778**	–0.151	–0.532	0.166	–0.133
SprintE	**0.842**	–0.201	–0.276	0.156	0.059	**0.798**	–0.258	0.305	–0.208	–0.022
SprintD	**0.817**	–0.194	–0.276	0.171	0.089	**0.734**	–0.273	0.363	–0.201	–0.056
HSRE	**0.945**	–0.182	–0.042	0.025	–0.054	**0.941**	–0.210	0.005	–0.050	0.037
HSRD	**0.944**	–0.178	–0.060	0.005	–0.013	**0.939**	–0.206	0.038	–0.044	0.010
HIRE	**0.960**	–0.193	–0.097	0.056	–0.030	**0.953**	–0.230	0.070	–0.087	0.026
HIRD	**0.959**	–0.193	–0.130	0.056	0.017	**0.946**	–0.240	0.135	–0.093	–0.008
MSRD	**0.834**	–0.163	0.295	–0.054	–0.121	**0.849**	–0.146	–0.226	0.124	0.014
LSRD	0.313	–0.091	**0.844**	–0.072	0.042	0.360	–0.058	–**0.761**	0.226	–0.214
**Eigenvalues**										
Total	7.128	4.416	1.787	1.385	1.092	6.916	4.461	1.777	1.313	1.259
% of variance	33.9	21.0	8.5	6.6	5.2	32.9	21.2	8.5	6.3	6.0
Cumulative%	33.9	55.0	63.5	70.1	75.3	32.9	54.2	62.6	68.9	74.9
**Bartlett’s test of sphericity**										
χ2	5521.94					5517.76				
p	< 0.001					< 0.001				
**KMO measure of sampling adequacy**	00.67					0.66				

**Bold represent loadings greater than ± 0.60.** TD, Total Distance; SprintD, Sprint Distance; SprintE, Sprint Efforts; HSRD, High-speed running distance; HSRE, High-speed running efforts; HIRD, High-intensity running distance; HIRE, High-intensity running efforts; MSRD, Moderate-speed running distance; LSRD, Low-speed running distance; ShotAcc, Shot Accuracy; BP, ball possession; PassAcc, Pass Accuracy; Fpass, Forward Passes; FPAcc, Forward Pass Accuracy; ChallengeW, Challenge Won.

**FIGURE 1:**
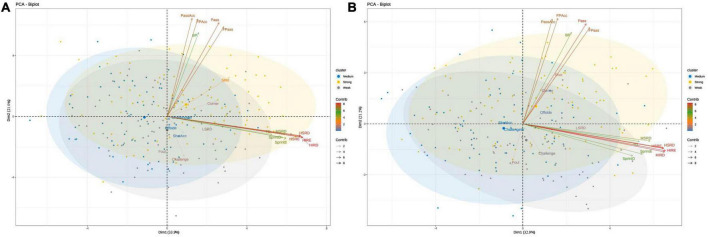
Principal component analysis (PCA) biplot of individuals and explanatory variables at home **(A)** and away matches **(B)**. The biplot shows the PCA scores of the explanatory variables as vectors and individuals among the three levels of teams in a two-dimensional space. Individuals on the same side as a given variable should be interpreted as having a high contribution on it. The magnitude of the vectors (lines) shows the strength of their contribution to each PC. The angle between the lines approximates the correlation between the explanatory variables they represent. The closer the angle is to 90, or 270 degrees, the smaller the correlation while An angle of 0 or 180 degrees reflects a correlation of 1 or –1, respectively. Colored concentration ellipses (size determined by a 0.95-probability level) show the observations grouped by mark class. TD, Total Distance; SprintD, Sprint Distance; SprintE, Sprint Efforts; HSRD, High-speed running distance; HSRE, High-speed running efforts; HIRD, High-intensity running distance; HIRE, High-intensity running efforts; MSRD, Moderate-speed running distance; LSRD, Low-speed running distance; ShotAcc, Shot Accuracy; BP, ball possession; PassAcc, Pass Accuracy; Fpass, Forward Passes; FPAcc, Forward Pass Accuracy; ChallengeW, Challenge Won.

Figure 1 displays a Principal component analysis (PCA) biplot of individuals and explanatory variables at home (Figure 1A) and away matches (Figure 1B). PC1 presented a positive correlation with physical-related variables such as HIRD, HIRE, HSRD, and HSRE while PC2 was positively associated with the passing-related variables such as Pass, FPass, PassAcc, and FPAcc. As such, PC1 typically represents intense-play styles while PC2 represents possession-play styles at home and away matches, respectively.

**FIGURE 2:**
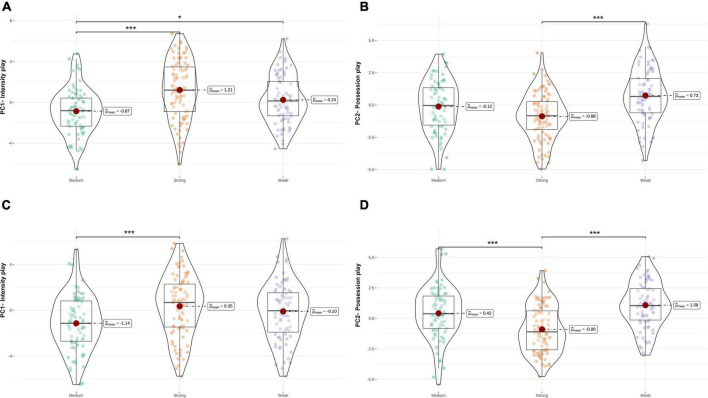
The differences among three levels of teams for both PCs at home **(A,B)** and away matches **(C,D)**.

Figure 2 shows the differences among three levels of teams for both PCs at home and away matches. There was an overall significant difference (see Figures 2A,B) at home matches between teams on PC1 (*F* = 15.173, *P* < 0.001, η^2^ = 0.115) and PC2 (*F* = 12.286, *P* < 0.001, η^2^ = 0.095). The pair-wise comparison showed a significant difference between strong and medium teams (*P* < 0.001, ES = 0.863), between medium and weak teams (*P* < 0.05, ES = 0.401) on PC1 (Figure 2A). Similarly, significant difference was found between strong and weak teams (*P* < 0.001, ES = 0.779) on PC2 (Figure 2B). In addition, there was an overall significant difference (see Figures 2C,D) at away matches between teams on PC1 (*F* = 9.368, *P* < 0.001, η^2^ = 0.074) and PC2 (*F* = 20.227, *P* < 0.001, η^2^ = 0.147). The pair-wise comparison showed a significant difference between strong and medium teams (*P* < 0.001, ES = 0.680) on PC1 (Figure 2C). Similarly, significant difference was found between strong and medium teams (*P* < 0.05, ES = –0.601), between strong and weak teams (*P* < 0.001, ES = 0.986) on PC2 (Figure 2D).

**FIGURE 3:**
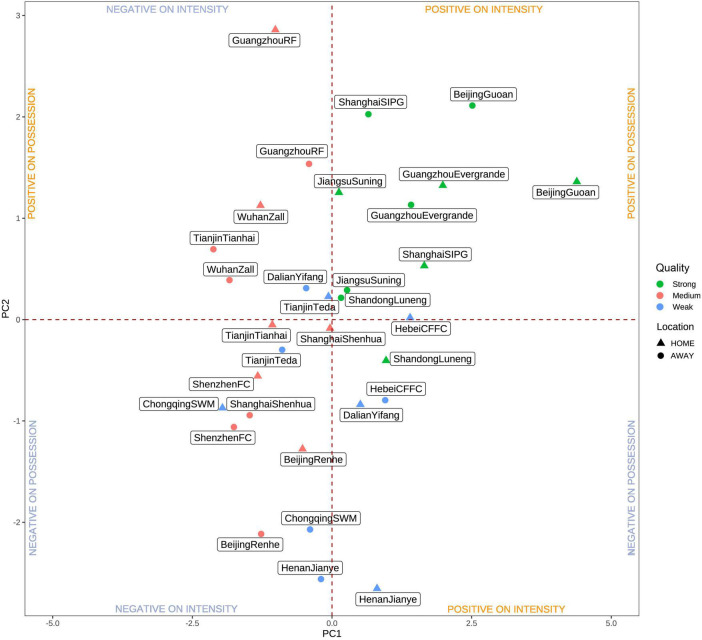
Combined graph of each team between home and away matches based on the principal components PC 1 and PC2 (X-axis dimension 1; Y-axis dimension 2).

The playing styles between home and away matches preferred by each team are presented in Figure 3. The first five teams in the final overall ranking in the CSL were located in the upper-right quadrant whereas the last five teams were located in the lower-left quadrant. The rest of the teams gathered around the origin of the coordinates. Specifically, Beijing Guoan and Shandong Luneng tended to utilize an intense-play style at home whereas they used a possession-play style at away. Furthermore, Guangzhou Evergrande presented balanced performances in terms of intensity and possession plays at home and away matches. In addition, Jiangsu Suning prefers to use more ball possession styles wherever they played, at home or away while Shanghai SIPG used the same style of play only at away matches. The team located in the lower-left quadrant represented the worst performance in terms of intensity and possession styles compared with other teams.

## Discussion

Our study provides novel evidence based on the previous study by adding physical and situational variables. In addition, the styles of play utilized by each team between home and away matches have shown different trends. Specifically, the first two components accounted for 55% of the total variance for home matches and 54.2% of the total variance for away matches. Our study noted the major significant contribution of PC1 included HIRD, HIRE, HSRD, and HSRE while PC2 mainly consisted of Pass, FPass, PassAcc, and PassAcc. Therefore, PC1 typically represented intensity-play styles while PC2 was associated with possession-play styles at home and away matches, respectively. In addition, strong teams preferred to utilize intensity play whereas medium and weak teams tended to be possession play when playing at home or away matches. Furthermore, the first five teams in the final ranking position in the CSL presented a compensated technical-physical playing style whereas the last five teams in the league showed inferior performance in terms of intensity and possession play. These investigations can provide coaches and managers a better understanding of competition patterns in the CSL to effectively improve the tactical and physical strategies when facing different opponents in their stadium or on the road.

Our study found that strong teams preferred to utilize high-intensity playing styles which are contradicted by Lago-Peñas et al. (2017) identified that top teams in the CSL preferred to maintain possession instead of giving the initiative to the opponent. A possible reason is that the previous study failed to consider the influence of physical-related variables on playing styles. In fact, the current study is in line with Zhou et al. (2021b) found that high-intensity and possession-play styles were considered the most important components of the playing patterns adopted by teams, and the overall trend showed an increase in terms of intensity play in the CSL. Indeed, top teams in the CSL preferred to play counter-attacking or direct play (Zhou et al., 2021a). For example, moving the ball quickly to within scoring range often utilized long passes or long balls downfield, which provides a higher requirement for high-intensity running and sprints (Gong et al., 2021). Furthermore, top teams within the CSL often recruit the best foreign players in different playing positions to activate more domestic players to participate in the overall offensive and defensive strategies which lead to accumulating distance at different ranges of velocity (Gai et al., 2019). Likewise, top teams could be required to maintain a high level of activity for players when not directly involved in play to create space to receive passes or to pressurize opponents into making mistakes in order to regain possession (Bradley et al., 2009; Gollan et al., 2020). Conversely, weak teams tended to perform more possession play in the CSL. The strategy of “maintaining possession” may involve more slow play with defensive movements, lower risk when passing, and greater emphasis on regaining possession relative to teams who might place less importance on this strategy (Lago, 2009; Fernandez-Navarro et al., 2018, 2019). This playing style is also called indirect play, which is slower than direct play and uses many short passes, while weaknesses in the opposition defense are sought (Lago, 2009; Gómez et al., 2018). In addition, our study found that high-intensity running with the aforementioned ball possession and passing ability is the key to a high ball possession strategy based on the first and second PC (Bradley et al., 2013). Therefore, coaches should design training tasks with continuous role changes, ensuring players concentrate to coordinate sudden movements with teammates from greater areas; and then improve positional decision-making due to a combination of a high number of ball controls, passes, and shots. Simultaneously, they need to perform many ball controls, passes, and shots during high-intensity aerobic endurance, combined with a higher number of accelerations and decelerations (Ade et al., 2016).

The first five teams in the final ranking are located in the upper-right quadrant, which indicates that these teams possess the best performance in terms of possession and intensity play (Miñano-Espin et al., 2017). Guangzhou Evergrande was the championship that won the league competition at the end of the season and its position in the chart was almost the same between home and away games suggesting that part of its success was associated with maintaining a balanced playing style and efficacy during the match-play. Beijing Guoan, Shandong Luneng, and Shanghai SIPG tended to employ intensity play at home matches whereas they used a possession play style at away matches. This result is in accordance with previous studies indicating that the tactical strategies of fast tempo, crossing, and high pressure in the offense phase were higher at home in comparison with away matches (Lago, 2009). These styles of play also were regarded as the aggressive play that aims to create as many scoring opportunities as possible and seems to be the overall trend when the team is playing at home (Castellano and Pic, 2019; Praça et al., 2021). Likewise, regaining ball possession in advanced zones of the pitch as a consequence of high-pressure strategies is linked to success (Almeida et al., 2014). Consequently, these results may support the influence of home advantage on playing styles in soccer. Although home advantage has been widely mentioned, the reasons remain unclear (Carron et al., 2005). Crowd support seems to be one of the major factors; however, referee bias, psychological factors, familiarity with the pitch, and travel impact seem to be the rest of the contributing factors (Nevill and Holder, 1999). Interestingly, Jiangsu Suning preferred to use more ball possession styles wherever they play, at home or away. Our result may further support the idea that when considering the effect of match location on technical and physical performances, the quality of the team and opponent should also be taken into account (Praça et al., 2021). In addition, teams with higher ball possession between home and away matches might utilize more set plays that increase the chance of obtaining a successful ball possession, especially when facing an intense defensive pressure situation (Bradley et al., 2013). On the other hand, the last five teams in the final ranking position are located in the lower-left quadrant, which indicates that these teams presented the worst performances in terms of possession and intensity play when playing at home or away. In addition, the rest of the teams are located in the upper-left or the lower-right quadrant where these teams present less compensated locations in the plot with a high predominance of components 2 or 1, respectively (Lopez-Valenciano et al., 2021). Collectively, intensity or possession play was associated with the final ranking positions in the CSL, and playing styles that combine these two factors could be more liable to win the competition (Lopez-Valenciano et al., 2021; Zhou et al., 2021b).

Some limitations need to be considered in future research. First, although the playing styles were identified for each team between home and away matches, the playing styles may be different which are subject to the policy and rules of the competition in their soccer league as well as the tactical strategies of each team (Lago-Peñas et al., 2017; Gómez et al., 2018). Second, playing styles may be adapted depending on several contextual factors such as match outcome, fixture congestion, and opposition quality (Fernandez-Navarro et al., 2018; Gollan et al., 2020). Future studies are recommended to consider the interactive effects of situational variables on playing styles based on the principal component analysis. Third, detailed analysis is required to determine match-to-match changes in playing style and efficacy variables in teams competing in the CSL.

## Conclusion

High-intensity and possession-play styles were considered the most important components of the playing patterns which were associated with the final overall ranking in the CSL. Strong teams preferred to utilize intensity play whereas medium and weak teams utilized possession play whenever playing at home or away matches. Furthermore, the first five teams in the final overall ranking in the CSL presented a compensated technical-physical playing style whereas the last five teams showed inferior performance in terms of intensity and possession play. Finally, playing styles that combine these two factors could be more liable to win the competition.

## Data availability statement

The original contributions presented in this study are included in the article/supplementary material, further inquiries can be directed to the corresponding authors.

## Author contributions

LK, SZ, M-AG, and CZ contributed to the conception and design of the study. CZ and SZ collected and organized the data. YH and TZ performed the statistical analysis. LK wrote the first draft of the manuscript. All authors contributed to the article and approved the submitted version.
